# Sharing of carbapenemase-encoding plasmids between Enterobacteriaceae in UK sewage uncovered by MinION sequencing

**DOI:** 10.1099/mgen.0.000114

**Published:** 2017-07-04

**Authors:** Catherine Ludden, Sandra Reuter, Kim Judge, Theodore Gouliouris, Beth Blane, Francesc Coll, Plamena Naydenova, Martin Hunt, Alan Tracey, Katie L. Hopkins, Nicholas M. Brown, Neil Woodford, Julian Parkhill, Sharon J. Peacock

**Affiliations:** ^1^​ London School of Hygiene and Tropical Medicine, London, UK; ^2^​ Wellcome Trust Sanger Institute, Cambridge, UK; ^3^​ Department of Medicine, University of Cambridge, Cambridge, UK; ^4^​ Clinical Microbiology and Public Health Laboratory, Public Health England, Cambridge University Hospitals NHS Foundation Trust, Cambridge, UK; ^5^​ Antimicrobial Resistance and Healthcare Associated Infections (AMRHAI) Reference Unit, National Infection Service, Public Health England, London, UK

**Keywords:** ESBL- producing Enterobacteriaceae, wastewater, sequencing, MinION

## Abstract

Dissemination of carbapenem resistance among pathogenic Gram-negative bacteria is a looming medical emergency. Efficient spread of resistance within and between bacterial species is facilitated by mobile genetic elements. We hypothesized that wastewater contributes to the dissemination of carbapenemase-producing Enterobacteriaceae (CPE), and studied this through a cross-sectional observational study of wastewater in the East of England. We isolated clinically relevant species of CPE in untreated and treated wastewater, confirming that waste treatment does not prevent release of CPE into the environment. We observed that CPE-positive plants were restricted to those in direct receipt of hospital waste, suggesting that hospital effluent may play a role in disseminating carbapenem resistance. We postulated that plasmids carrying carbapenemase genes were exchanged between bacterial hosts in sewage, and used short-read (Illumina) and long-read (MinION) technologies to characterize plasmids encoding resistance to antimicrobials and heavy metals. We demonstrated that different CPE species (*Enterobacter kobei* and *Raoultella ornithinolytica*) isolated from wastewater from the same treatment plant shared two plasmids of 63 and 280 kb. The former plasmid conferred resistance to carbapenems (*bla*_OXA-48_), and the latter to numerous drug classes and heavy metals. We also report the complete genome sequence for *Enterobacter kobei*. Small, portable sequencing instruments such as the MinION have the potential to improve the quality of information gathered on antimicrobial resistance in the environment.

## Abbreviations

AMRHAI, antimicrobial resistance and healthcare-associated infections; CPE, carbapenemase-producing Enterobacteriaceae; CUH, Cambridge University Hospitals NHS Foundation Trust; ENA, European Nucleotide Archive; IS, insertion sequence; MIC, minimum inhibitory concentration; MβL, metallo-β-lactamase; PacBio, Pacific BioSciences RSII system; SNP, single-nucleotide polymorphism; WGS, whole-genome sequencing.

## Data Summary

1. Illumina sequence data have been deposited in the European Nucleotide Archive (ENA); individual sample accession numbers are as listed in Table 1. MinION sequence data have been deposited in ENA; accession numbers: ERS634378, ERS634376 and ERS1033541 (www.ebi.ac.uk/ena/data/view/ERS634378, www.ebi.ac.uk/ena/data/view/ERS634376, www.ebi.ac.uk/ena/data/view/ERS1033541).

2. Supporting data, including assemblies and the fast52fastq.py script, are available from a GitHub repository (https://github.com/kim-judge/wastewater).

3. The manually finished genome of *Enterobacter kobei* has been deposited in ENA under accession numbers: FKLS01000001–FKLS01000010 (www.ebi.ac.uk/ena/data/view/FKLS01000001 – www.ebi.ac.uk/ena/data/view/FKLS01000010).

## Impact Statement

The spread of carbapenem resistance among pathogenic Gram-negative bacilli is a major public-health threat. Studies to date have emphasized this problem in the clinical setting, largely ignoring the importance of environmental contamination and the significance of a One Health approach. The use of Illumina whole-genome sequencing to identify the genetic basis for resistance led to the discovery that several species from the same treatment plant carried the same carbapenem-resistance gene. Using the Oxford Nanopore MinION, we demonstrated putative sharing of several plasmids between different Enterobacteriaceae species (*Enterobacter* *kobei* and *Raoultella ornithinolytica*) in wastewater, with one plasmid carrying a carbapenemase gene conferring resistance to the carbapenem drugs, and the other containing resistance genes to numerous other drugs and heavy metals. To our knowledge, this is the first report of carbapenemase-producing Enterobacteriaceae being recovered from wastewater at UK sewage treatment plants. Importantly, we show that treatment plants do not prevent the release of these bacteria into the environment and this may provide a point of intervention. We also demonstrate the utility of a next-generation sequencing instrument, the MinION, to confirm the sharing of plasmids between bacterial isolates in wastewater, and have generated a complete genome sequence for *Enterobacter kobei*. The ease of use of this instrument would facilitate its implementation in future intervention studies that target environmental contamination with carbapenem-resistance genes.

## Introduction

The global rise of carbapenemase-producing Enterobacteriaceae (CPE) over the last decade represents a major threat to public health [1–3]. CPE are often resistant to several additional classes of antibiotics, which may limit therapeutic options to drugs with a higher toxicity profile. Unsurprisingly, invasive infections caused by CPE are associated with poor clinical outcomes and carry an excess attributable mortality compared with those caused by carbapenem-susceptible isolates [4–6]. CPE are predominantly isolated in healthcare settings, but their spread to healthy humans, livestock and the environment has been reported [4, 7]. The development of interventions that prevent further CPE dissemination requires delineation of their reservoirs and routes of spread, combined with accurate characterization of the mobile elements that transfer carbapenemase genes within and between bacterial species.

High-throughput whole-genome sequencing (WGS) has provided major insights into pathogen transmission and outbreak investigation based on core-genome phylogenetic analyses [8], but has not reached its potential to provide detailed characterization of plasmids and their transmission within specific environments. This is due to a methodological limitation of short-read data generated by Illumina technology, from which plasmids cannot be accurately assembled because of the presence of numerous repetitive regions. Technologies including the Pacific BioSciences RSII system (PacBio) and the Oxford Nanopore Technologies MinION system generate long-read data that can overcome this barrier. It has been demonstrated previously that PacBio long-read sequencing can be successfully used to elucidate transfer of carbapenem-resistance-carrying plasmids within hospital environments [9, 10], and after years of on-going development the pocket-sized MinION is approaching a level of accuracy and usability at which it could be used to explore the central question of plasmid transmission. It has been used previously to detect the presence of antimicrobial-resistance genes and to track the transmission of viral outbreaks [11–14].

Our aim here was to apply the MinION and Illumina platforms to seek evidence for sharing of carbapenemase-encoding plasmids between bacterial species in human sewage. This reservoir contains a diversity of pathogenic and non-pathogenic bacteria that can exchange DNA (including genes encoding antimicrobial resistance) by horizontal gene transfer. CPE have been isolated from human sewage at wastewater treatment plants in Austria, Germany and Brazil, and from rivers and lakes in Switzerland, Portugal, Brazil and Vietnam [15–22], and carbapenemase-encoding plasmids have been found in wastewater in Germany [21], but definitive evidence of plasmid sharing and putative transmission is lacking.

**Table 1.** CPE isolated from wastewater and their antimicrobial-resistance mechanisms

						Genetic mechanisms derived from sequence data			
Wastewater treatment plant	Untreated or treated sample	Isolate ID	Accession no.	Species	Phenotypic carbapenem- resistance mechanism	Carbapenem resistance*	Extended-spectrum β-lactamases	Narrow-spectrum β-lactamases	Other resistance genes
W1	Treated	VRES0316	ERS634377	*Escherichia coli*	MβL	*bla*_NDM-5_	–	–	Macrolide (*mphA*); sulphonamide (*sul1*); trimethoprim (*dfrA12*)
W2	Untreated	VRES0375	ERS634381	*Klebsiella pneumoniae*	MβL	*bla*_NDM-1_-like	–	*bla*_TEM-1_, *bla*_OXA-1_, *bla*_LEN-25_-like*	Aminoglycoside [*aac(6′)-Ib-cr*, *aac(3)-IIa*, *strA/B*]; chloramphenicol (*catA1*); fosfomycin (*fosA*); quinolone (*oqxA, oqxB, qnrB1*); sulphonamide (*sul1*); tetracycline [*tet*(A)]; trimethoprim (*dfrA12, dfrA14*)
W2	Untreated	VRES0377	ERS808629	*Enterobacter cloacae* complex	MβL	*bla*_IMP-1_	–	–	Aminoglycoside (*aac(6′)-Ib*, *aadB*); fosfomycin (*fosA*); macrolide (*ereA*); quinolone (*qnrA1*); sulphonamide (*sul1*); trimethoprim (*dfrA1*)
W2	Untreated	VRES0379	ERS808630	*Klebsiella oxytoca*	OXA-48-like	*bla*_OXA-48_	*bla*_CTX-M-15_, *bla*_OXY-6_	*bla*_TEM-1_, *bla*_OXA-1_	Aminoglycoside [*aac(6′)-Ib-cr*, *aac(3′)-IIa, strA, strB*]; trimethoprim (*dfrA14*); quinolone (*qnrB1*); sulphonamide (*sul2*); tetracycline [*tet*(A)]
W3	Untreated	VRES0380	ERS634382	*Klebsiella pneumoniae*	OXA-48-like	*bla*_OXA-181_ and *ompF*†	*bla*_CTX-M-15_, *bla*_SHV-12_, *bla*_CMY-4_	*bla*_TEM-1_	Aminoglycoside [*aac(6′)-Ib-cr, armA*]; fosfomycin (*fosA*); macrolide (*ermB, mphE, msrE*); quinolone (*oqxA, qnrS1*); rifampicin (*arr-2*); sulphonamide (*sul1*); trimethoprim (*dfrA12*)
W4	Untreated	VRES0269	ERS634376	*Raoultella ornithinolytica*	OXA-48-like	*bla*_OXA-48_	*bla*_SHV-12_	*bla*_TEM-1_	Aminoglycoside [*aac(6′)-Ib*, *aac(6′)-IIc*]; fosfomycin (*fosA*); quinolone (*qnrA1*); rifampicin (*arr*); sulphonamide (*sul1, sul2*)
W4	Untreated	VRES0273	ERS634378	*Enterobacter kobei*	OXA-48-like	*bla*_OXA-48_ and *ompCF*‡	*bla*_SHV-12_	*bla*_TEM-1_	Aminoglycoside [*aac(6′)-IIc*]; fosfomycin (*fosA16*); quinolone (*qnrA1*); rifampicin (*arr*); sulphonamide (*sul1, sul2*)
W4	Treated	VRES0259	ERS1033541	*Raoultella ornithinolytica*	OXA-48-like	*bla*_OXA-48_	*bla*_SHV-12_, *bla*_OXA-17_	*bla*_TEM-1_	Aminoglycoside [*aac(6′)-Ib*, *aac(6′)-IIc*]; fosfomycin (*fosA*); quinolone (*qnrA1*); rifampicin (*arr*); sulphonamide (*sul1, sul2*)
Cambridge hospital sewer	Untreated	VRES0183	ERS634380	*Klebsiella pneumoniae*	KPC	*bla*_GES-5_ and *ompCF§*	*bla*_CTX-M-15_, *bla*_SHV-12_	*bla*_OXA-1_, *bla*_TEM-1_	Aminoglycoside [*aac(6′)-Ib*, *aac(6′)-Ib-cr*, *aac(3)-IIa*, *aph(3)-Ia*]; fosfomycin (*fosA*); macrolide (*mphA*); quinolone (*oqxA*, *oqxB*); tetracycline [*tet*(A), *tet*(D)]

*Genes labelled as ‘-like’ differ from the named gene by 1 aa change.

†VRES0380 – *ompF* frameshift mutation.

‡VRES0273 – *ompC* insertion of IS element, *ompF* introduction of stop codon.

§VRES0183 – *ompC* introduction of stop codon and potentially IS element insertion, *ompF* frameshift mutation.

## Methods

### Wastewater processing and bacterial identification

Samples of treated and untreated wastewater were obtained from each of 20 treatment plants. At each sampling point, two consecutive grab samples of 0.5 l each were collected and mixed in 1 l sterile bottles containing 18 mg sodium thiosulphate (Sigma-Aldrich). A single 1 l wastewater sample was obtained from the main septic tank at the Cambridge University Hospitals NHS Foundation Trust (CUH) facility. All samples were transported to the laboratory on ice packs in the dark and processed within 12 h. One millilitre of triplicate serial tenfold dilutions, 10 ml untreated wastewater samples and 100 ml treated wastewater samples were concentrated using the filtration technique onto 0.45 µm pore size filter membranes (S-Pak; Merck Millipore). Membranes were then placed onto the surface of ESBL *Brilliance* agar (Oxoid) and incubated for 24 h at 37 °C in air. Duplicate membranes of the lowest dilution tested were also placed into tryptic soy broth with 2 mg imipenem l^−1^ and incubated for 24 h at 37 °C in air, followed by subculture of 100 µl on chromID CARBA SMART plates (bioMérieux), and ESBL *Brilliance* and cystine lactose electrolyte deficient (CLED) agar with a 10 µg imipenem disc.

At least one colony for each bacterial colony morphology type suspected to be Enterobacteriaceae based on colour were picked and speciated using matrix-assisted laser desorption/ionization-time of flight mass spectrometry (MALDI-TOF MS; Biotyper version 3.1; Bruker Daltonics). Antimicrobial-susceptibility testing was determined using the N206 card on the Vitek 2 instrument (bioMérieux) calibrated against European Committee on Antimicrobial Susceptibility Testing (EUCAST) breakpoints. Minimum inhibitory concentrations (MICs) of meropenem, ertapenem, imipenem and colistin were determined using Etest (bioMérieux) for any isolate with reduced susceptibility to carbapenems on Vitek 2. Enterobacteriaceae with confirmed resistance to any carbapenem underwent further phenotypic testing using the KPC/MBL and OXA-48 Confirm kit (Rosco Diagnostica) to detect production of KPC, MBL and OXA-48 carbapenemases. All CPE (*n*=16) identified were de-duplicated for species and type of carbapenemase according to sample location, and further analysed using WGS (*n*=9).

### Illumina sequencing and bioinformatic analyses

DNA extraction and library preparation were performed as previously described [23]. DNA libraries were sequenced using the Illumina HiSeq and MiSeq platforms (Illumina) to generate 100 and 150 bp paired-end reads, respectively. *De novo* multi-contig draft assemblies were generated using Velvet Optimiser and Velvet [24]. Contigs smaller than 300 bases were removed, the scaffolding software sspace was employed, and assemblies further improved using GapFiller. For isolates belonging to the *Enterobacter cloacae* complex, species identification was based on analysis of *hsp60* and *rpoB*, as described elsewhere [25]. To detect acquired genes encoding antimicrobial resistance, a manually curated version of the ResFinder database (compiled in 2012) [26] was used. Assembled sequences were compared to this as described previously [27], and genes with 100 % match to length and >90 % identity match were classified as present and variants identified. To establish genetic relatedness between *Raoultella ornithinolytica* isolates, Illumina sequence reads were mapped to the reference genome B6 (GenBank accession no. CP004142) using smalt v0.7.4 to identify single-nucleotide polymorphisms (SNPs). SNPs were filtered to remove those at sites with a SNP quality score below 30, and SNPs at sites with heterogeneous mappings were filtered out if the SNP was present in less than 75 % of reads at that site. Sequence data have been deposited in the European Nucleotide Archive (www.ebi.ac.uk/ena) under the individual accession numbers given in Table 1.

### MinION sequencing and bioinformatic analyses

Isolates were streaked from frozen stock onto blood agar plates and incubated in air at 37 °C overnight. A sweep of colonies was taken and DNA extraction carried out using the QiaAMP DNA mini kit (Qiagen) and quantified using the Qubit fluorometer and BR kit (Life Technologies). Genomic DNA was diluted in 10 mM Tris-HCl to a concentration of 1500 ng in 46 µl and sheared using G-TUBES (Covaris). MinION sample preparation and two-dimensional sequencing was carried out as previously described using the SQK-MAP-006 kit and R7.3 flow cells [28]. Basecalling was carried out using the Metrichor two-dimensional basecalling option using version 1.15.7 for VRES0269 and VRES0273, and version 1.20.5 for VRES0259.

Basecalled MinION reads were converted from fast5 to fastq and fasta formats using fast52fastq.py (https://github.com/kim-judge/wastewater). Read mapping was carried out to assess the quality of data and coverage using the bwa-mem algorithm of bwa v0.7.12 with the flag –x ont2d [29]. Output sam files from bwa-mem were converted to sorted bam files using SAMtools v0.1.19-44428 cd [30]. Assembly using MinION data only was undertaken using Canu [31, 32], followed by Circlator [33] 1.2.0. Canu version 1.0 was run using the commands maxThreads=8, maxMemory=16, useGrid=0, nanopore-raw. Hybrid assemblies were generated using SPAdes 3.6 [34] using the –nanopore and –careful flags, then filtered to exclude contigs of less than 1 kb. All plasmid sequences and the chromosomes were confirmed to be circular by the Circlator software 1.2.0. Assembly statistics were analysed using assembly-stats (https://github.com/sanger-pathogens/assembly-stats). All assemblies were annotated using Prokka 1.5 [35, 36]. Assemblies were compared using the Artemis comparison tool [37] and further analysed using Artemis [38]. Plasmid sequences and other regions of interest were investigated using the blastn suite at National Center for Biotechnology Information (http://www.ncbi.nlm.nih.gov).

### Manually finished genome

Assemblies were generated using Canu and SPAdes as described above. A gap5 database was made using corrected MinION pass reads from the Canu pipeline and the Illumina reads. Manual finishing was undertaken using gap5 [39] version 1.2.14 making one chromosome and nine plasmids. Icorn2 [40] was run on this for five iterations. The start positions of the chromosome and plasmids were fixed using Circlator [33] 1.2.0 using the command circlator fixstart. This assembly was annotated using Prokka 1.5 [35, 36].

### Regional surveillance of CPE

Information was retrieved on all CPE isolates referred from diagnostic microbiology laboratories in the East of England to Public Health England’s Antimicrobial Resistance and Healthcare Associated Infections (AMRHAI) Reference Unit between 2006 and 2015 (Table S1, available in the online Supplementary Material). KPC-, OXA-48-like-, NDM-, VIM- and IMP-encoding genes were detected by in-house PCR and/or commercial microarray, which was performed by the AMRHAI Reference Unit using published methods [41–46].

## Results

### Isolation of CPE from wastewater

**Fig. 1.**
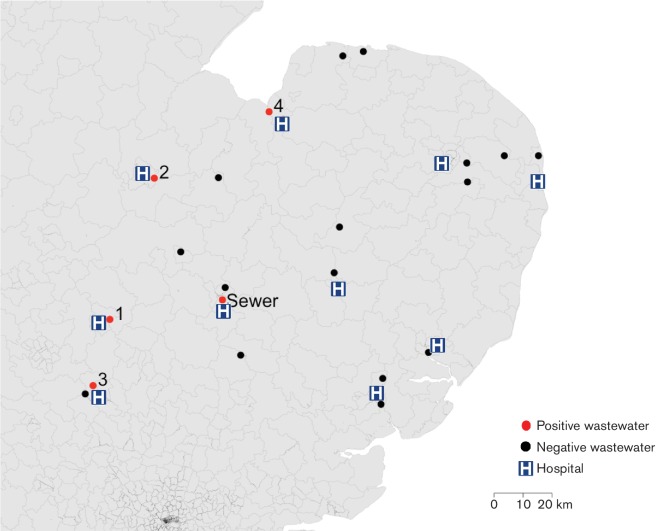
Map of wastewater treatment plants in the East of England tested for CPE. Black dots, negative for CPE; red dots, positive for CPE. Plants positive for CPE are numbered 1–4. Sewer refers to sampling at the CUH facility. Hospitals situated upstream of the study wastewater treatment plants (approximate median distance of 5.3 km; range 3.3–9.6 km) are denoted by H.

We undertook a cross-sectional study of 20 municipal wastewater treatment plants in the East of England between June 2014 and January 2015. Plant location was selected with reference to hospital waste, with ten plants situated immediately downstream of acute NHS Hospital Trust facilities (approximate median distance between wastewater treatment plant and respective hospital 5.3 km, range 3.3–9.6 km) and ten plants not connected to acute hospital effluent (Fig. 1). Paired samples of untreated and treated wastewater were obtained from each plant. The main septic tank at the CUH facility was also sampled in September 2014. CPE were isolated using filtration and culture procedures (see Methods).

CPE were isolated from four plants and the CUH sewer (see Fig. 1 for locations). All four positive plants directly received hospital effluent. A total of nine bacterial isolates belonging to six species were cultured, all of which may cause human infection (Table 1). *E. coli* (*n*=1) and *R. ornithinolytica* (*n*=1) were isolated from treated water that was destined for release into the environment, and *Klebsiella pneumoniae* (*n*=3)*, Klebsiella oxytoca* (*n*=1)*, Enterobacter cloacae* complex (*n*=2) and *R. ornithinolytica* (*n*=1) were isolated from untreated water. In one case, the same species (*R. ornithinolytica*) was isolated from pre- and post-treated water sampled from the same plant.

### Phenotypic evaluation of antimicrobial resistance

To characterize the carbapenemases in the nine isolates, we first used a phenotypic method that detects the three main biochemical groups of carbapenemases in Enterobacteriaceae. This identified a class A serine carbapenemase in *K. pneumoniae*, class B metallo-β-lactamases (MβLs) in *E. coli*, *K. pneumoniae* and *Enterobacter cloacae* complex, and class D OXA-48-like carbapenemases in *Enterobacter cloacae* complex, *K. pneumoniae*, *K. oxytoca* and the two *R. ornithinolytica* (Table 1). Susceptibility testing was conducted against a panel of antimicrobials, and demonstrated that all nine isolates were resistant to at least five antibiotic drug groups (Table 2).

**Table 2.** Antimicrobial susceptibility of CPE isolated from wastewater

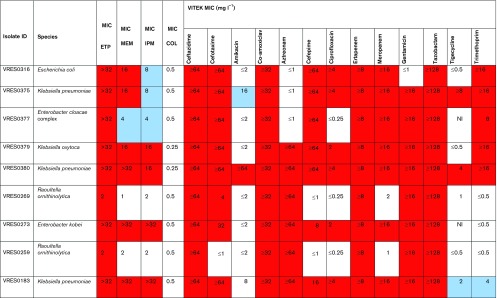

COL, Colistin; ETP, ertapenem; IPM, imipenem; MEM, meropenem; ni, non-identifiable by VITEK.

Red shading, resistant; blue shading, intermediate resistance; white/no shading, susceptible.

### Comparison between wastewater findings and geographically related clinical isolates

**Fig. 2.**
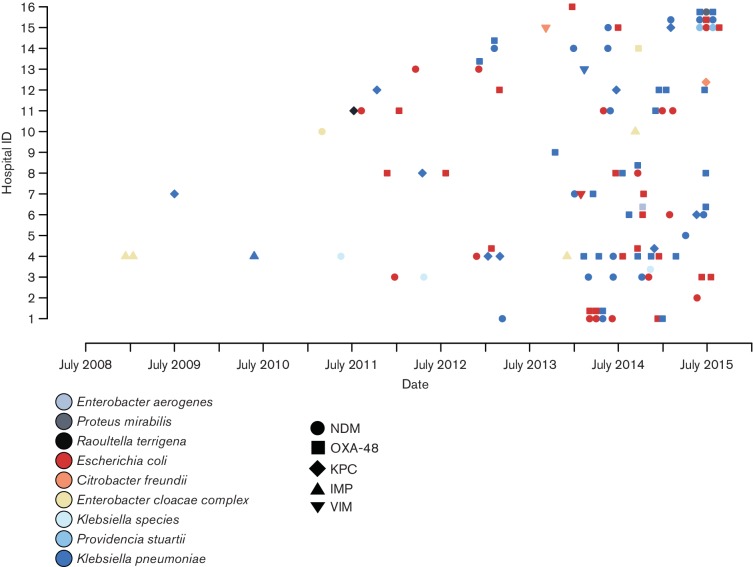
CPE isolates referred from diagnostic microbiology laboratories in the East of England to the AMRHAI Reference Unit between 2008 and 2015. One representative of the same CPE per month per hospital is shown. One sample received by PHE in 2006 was excluded as date of isolation was unknown. See Table S1 for a complete list of all isolates.

To compare our findings with carbapenem-resistance mechanisms present in isolates cultured in the clinical setting in the same region, we collated data gathered by Public Health England on all CPE isolates referred by 16 diagnostic microbiology laboratories in the East of England to their AMRHAI Reference Unit for further characterization between 2006 and 2015. This identified 115 CPE isolates belonging to nine different Enterobacteriaceae species and harbouring carbapenemases belonging to classes A, B and D (Table S1). *K. pneumoniae*, *Escherichia coli* and members of the *Enterobacter cloacae* complex accounted for 43, 38 and 8 % of isolates, respectively. The same bacterial species and carbapenemase gene (NDM) was observed in 2 wastewater treatment plants (numbered 1 and 2, Fig. 2) located downstream of hospitals where such CPEs were also identified.

### Genetic characterization of antimicrobial resistance

The entire repertoire of resistance genes/mechanisms was characterized in the nine study isolates by performing WGS using Illumina short-read technology, followed by comparison of each genome to a comprehensive database of genes/gene variants that encode drug resistance (See Methods for details). From this, we identified carbapenemases in class A (GES-5), class B [NDM-5, NDM-1-like (differed from NDM-1 by 1 aa change) and IMP-1] and class D (OXA-48 and OXA-181) (Table 1), which are known to be carried by mobile genetic elements. Three isolates with high imipenem resistance also had truncated versions of porin proteins due to insertion sequences, stop mutations and frameshift mutations identified in ompC and ompF genes (Table 1). Disruption of these genes have been associated previously with high levels of carbapenem resistance [47]. Genes conferring resistance to multiple classes of antimicrobials were identified in all nine isolates, with gentamicin, quinolone and sulphonamide resistance being the most prevalent (Table 1).

Three CPE isolates were recovered from the same treatment plant: *Enterobacter cloacae* complex VRES0273 and *R. ornithinolytica* VRES0269 from pre-treated water, and *R. ornithinolytica* VRES0259 from post-treated water. VRES0273 was identified as *Enterobacter kobei* based on analysis of *hsp60* and *rpoB*, as described elsewhere [25, 48]. To assess the core genetic relatedness of the two *R. ornithinolytica* isolates, we aligned Illumina reads from both against the reference genome *R. ornithinolytica* B6 [49]. This identified that both genomes were over 18 000 SNPs different from the reference over a core genome length of 5 398 151 bp (equating to 99.7 % identity), but only 13 SNPs different between each other. This indicates a very high degree of relatedness. In addition, both *R. ornithinolytica* isolates had identical antibiotic-resistance patterns for 14/15 antimicrobials tested (Table 2). The three CPE isolates from this plant all contained the *bla*_OXA-48_ gene conferring carbapenem resistance and the same non-β-lactam-resistance genes (Table 1).

### Sharing of resistance plasmids between Enterobacteriaceae in wastewater

**Fig. 3.**
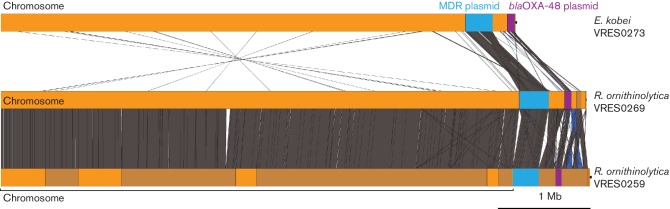
Comparison of whole-genome assemblies of *Enterobacter kobei* and *R. ornithinolytica* isolates using the Artemis comparison tool. The grey and dark blue vertical blocks represent blast hits between the isolates in the same orientation or in the inverted orientation, respectively. Only hits longer than 1 kb are shown. Contigs are highlighted in alternating orange and brown colours. The MDR plasmid is highlighted in light blue, and the *bla*_OXA-48_ plasmid is highlighted in purple.

We hypothesized that resistance was mediated by one or more plasmids that were shared between these three isolates. We first examined the Illumina data alone to determine whether a plasmid carrying the *bla*_OXA-48_ gene was the same in the three isolates, but predictably we failed to assemble complete plasmid sequence from this short-read data. From this, we were unable to conclude whether plasmids were shared, nor their specific content. The *bla*_OXA-48_ gene was located on contigs of 2.5 kb (VRES273), 3.6 kb (VRES269) and 3.4 kb (VRES259) in the Illumina assemblies. To overcome this, DNA from the three isolates was sequenced using the MinION system to generate long-read data and assembled as described in Methods. From this we assembled a manually finished genome sequence for *Enterobacter kobei* (VRES0273), which represents the first complete whole-genome sequence for this species, and high-quality draft assemblies for the two *R. ornithinolytica* isolates. Tables 3 and S2 summarize the Illumina and MinION sequencing data. Using these assemblies, we were able to confirm the close genetic relationship between the two *R. ornithinolytica* isolates by mapping each against the other. These were 12 SNPs different over 5 344 247 aligned bases in a chromosome of 5 462 249 bp (VRES0269) and 5 334 936 aligned bases in a chromosome of 5 392 238 bp (VRES0259), corresponding to 99.9 % identity in both cases, and covering 97.8 and 98.9 % of the genome, respectively. One large genomic difference was present between the two isolates due to the absence of one of the four phages in VRES0259 (Fig. 3).

**Table 3.** Assembly statistics for the nine CPE isolates based on Illumina and MinION data

Isolate ID	Species	Accession no.	Coverage	No. of contigs	Genome size [bp]	N50 [bp]*	Illumina or MinION data
VRES0316	*E. coli*	ERR885454	91×	104	5 123 602	197 358	Illumina
VRES0375	*K. pneumoniae*	ERR885458	72×	114	6 264 863	220 223	Illumina
VRES0377	*Enterobacter cloacae* complex	ERR1100748	72×	64	5 499 401	293 804	Illumina
VRES0379	*K. oxytoca*	ERR1100749	52×	112	6 676 582	186 596	Illumina
VRES0380	*K. pneumoniae*	ERR885459	88×	57	5 657 356	430 982	Illumina
VRES0269	*R. ornithinolytica*	ERR885453	73×	99	6 178 860	235 729	Illumina
		ERR1341571 ERR1341570	35×	14	6 270 467	3 218 273	MinION
		na	na	9	6 345 266	5 614 685	Both
VRES0273	*Enterobacter kobei*	ERR885455	75×	90	5 454 767	153 115	Illumina
		ERR1341575 ERR1341574	42×	15	5 542 520	2 782 732	MinION
		na	na	13	5 576 147	5 303 011	Both
VRES0259	*R. ornithinolytica*	ERR1539195	654×	79	6 179 725	293 454	Illumina
		ERR1341573 ERR1341572	65×	na†	na†	na†	MinION
		na	na	19	6 399 880	1 238 878	Both
VRES0183	*K. pneumoniae*	ERR885457	80×	68	5 544 688	300 916	Illumina

na, Not applicable.

*N50 is a weighted median statistic. Half (50 %) of the assembly is contained in contigs greater than or equal to a contig of this size.

†There is no MinION only assembly for VRES0259 as the data for this isolate was only sufficient to make a hybrid assembly.

**Fig. 4.**
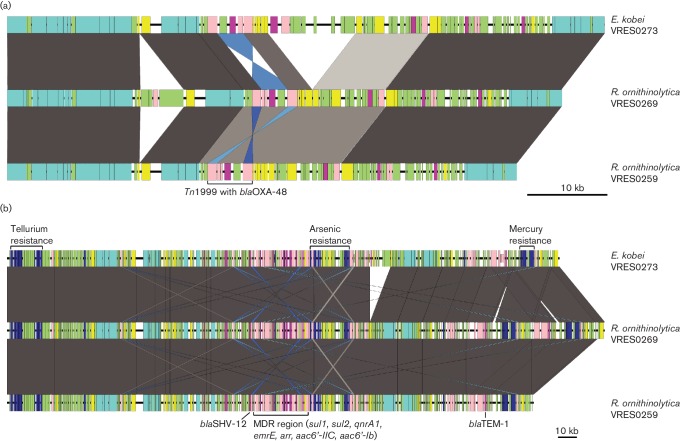
Comparison of shared *Enterobacter kobei* and *R. ornithinolytica* plasmids. (a) *bla*_OXA-48_ pOXA-48a-like plasmid. (b) Multidrug-resistance plasmid. Plasmid maps of the shared plasmids are shown, with genes of interest annotated. The grey and blue blocks represent blast hits between the isolates in the same orientation and inverted orientation, respectively. The colour intensity is proportional to the per cent identity of the match, within the specific region. Gene colour code indicates function: dark blue, heavy-metal resistance; light blue, conjugational transfer; dark pink, antibiotic resistance; light pink, IS elements and transposases; yellow, replication, maintenance, partitioning genes; light green, other (hypothetical proteins, host metabolism, regulators and pseudogenes).

One contig from each isolate (74 kb in *Enterobacter kobei*, 69 kb in *R. ornithinolytica* VRES259 and 63 kb in *R. ornithinolytica* VRES269) contained the *bla*_OXA-48_ gene (Fig. 4a). Based on blast searches and assembly comparisons using the Artemis comparison tool, these contigs showed synteny and orthology to other widespread IncL/M-type *bla*_OXA-48_ plasmids in *Enterobacter cloacae, R. ornithinolytica* and *K. pneumoniae* (GenBank examples: JN626286.1, KP061858.1, NC_023027.1, CVRH01000036 to CVRH01000038 and LN864819.1) [41, 50–54]. The plasmids from the three isolates were identical to each other over a shared region of 63 kb, with zero SNPs difference. Variation in overall size was due to hypothetical proteins integrated via insertion sequence (IS) elements. There were several transposases, which were located around the *bla*_OXA-48_ gene and forming part of the Tn*1999* transposon (Fig. 4a). Similar variation in plasmids encoding other carbapenemases has been identified in isolates from different species at a single hospital [9, 10].

We also identified a 297 kb contig in *Enterobacter kobei*, a 318 kb contig in *R. ornithinolytica* VRES259 and a 281 kb contig in *R. ornithinolytica* VRES269 from long-read data (Fig. 3). All three contigs were identified as an IncHI2 type plasmid using *in silico* PCR [55]. These carried heavy-metal-resistance genes for mercury, tellurium and arsenic, and conjugational transfer genes that facilitate plasmid transfer between different species of Enterobacteriaceae (Fig. 3b). A multidrug-resistance region similar to those found in *Enterobacter cloacae* [56] (GenBank examples: CP012170.1, EU855788.1, CP008899.1) was also identified in this plasmid, which contained resistance genes for aminoglycosides [(aac(6′)*-IIc*, aac(6′)*-Ib*), ethidium bromide (*emrE*), rifampicin (*arr2*), quinolones (*qnrA1*), sulphonamides (*sul1*, *sul2*) and third-generation cephalosporins (*bla*_SHV-12_)]. This multidrug-resistance region was flanked by transposases, indicating the potential for excision and horizontal gene transfer (Fig. 4b). The shared plasmid had a common 280 kb region, which contained three SNPs between the *Enterobacter kobei* plasmid and the other two plasmids, and zero SNPs between the *R. ornithinolytica* plasmids.

## Discussion

To our knowledge, this is the first report of CPE being recovered from wastewater at UK sewage treatment plants. Our demonstration of the co-circulation of a diversity of pathogenic, carbapenem-resistant Gram-negative species carrying different resistance genes in sewage confirms that this is a complex and diverse reservoir. We also confirmed that treatment processes do not prevent the release of CPE into the environment, which will contribute to contamination of river and lakes, farmland, vegetable land and fisheries, with the potential for spread to humans and livestock. Human infection caused by bacteria such as those identified here would prove challenging to treat with available antimicrobial drugs.

All CPE isolates were recovered from plants located downstream of hospitals. The same bacterial species and carbapenemase gene (encoding NDM) was found in two hospitals and the downstream wastewater treatment plant, indicating that the resistance mechanisms found in wastewater are likely to mirror those associated with human disease. Currently, no national regulation nor the European Directive 91/271/EEC on urban wastewater treatment stipulates the use of disinfection of hospital wastewater in the UK, and as a result this may be contributing to the dissemination of CPE [57, 58]. Further studies are required to fully ascertain the role of hospital effluent as a source of environmental contamination with CPE.

Phenotypic methods presumptively identify the presence of the most common CPEs (KPC, MBL, OXA-48), but are unable to identify variant types. We used genome sequencing to identify variants of carbapenemase-resistance genes, to detect sequence alterations in porin genes associated with decreased membrane permeability (an important factor contributing to carbapenem resistance [59]), and to define the full repertoire of resistance genes. The use of long-read sequencing data enabled us to conclude that two Enterobacteriaceae species (*R. ornithinolytica* and *Enterobacter kobei*) in treated and untreated wastewater carried highly similar plasmids containing numerous genes encoding resistance to antimicrobials and heavy metals. Whilst the rate of mutation in plasmids may vary from that of the chromosome, our findings are consistent with the suggestion that a globally distributed *bla*_OXA-48_ plasmid was shared very recently between the two species (0 SNPs different), a figure that is contextualized by comparison with a published sequence of plasmid pOXA-48a [50], which was more than 100 SNPs different. This plasmid is known to be the origin of the widespread dissemination of *bla*_OXA-48_ and, similar to our findings, has a broad bacterial host range and does not encode additional antimicrobial-resistance genes [50, 60]. In addition, a recent report from Findlay *et al*. in 2017 [3] identified OXA-48-like enzymes as being the second most frequently detected carbapenemases in the West Midlands region of the UK between 2007 and 2014 based on a study of 119 clinical isolates, including one environmental isolate from an endoscope camera head in a urology theatre. OXA-48-like enzymes were identified in 16/119 isolates from three different bacterial species (*K. pneumoniae*, *E. coli* and *Citrobacterfreundii*). Similar to the plasmids detected in our study, five IncL/M plasmids were identified using WGS, which exhibited >99 % identity to pOXA-48a with no additional resistance genes. These genome data were not publicly available and no genome comparison was possible with our data, but based on similarity to pOXA-48a and genetic content we postulate that a highly related plasmid encoding *bla*_OXA-48_ is found in both UK wastewater and associated with human infection. Similar to pOXA-48a, the *bla*_OXA-48_ gene and the transposases identified in our study are part of the Tn*1999* transposon, indicating the mobility of this gene independent of the plasmid backbone (Fig. 4a). It has also been shown in an early characterization of this plasmid that it is highly identical and syntenic to the pCTX-M3 plasmid, except for the Tn*1999* transposon from which it might have evolved [50].

The multidrug and heavy-metal resistance plasmid was highly similar in both species, although this acquisition may have occurred less recently based on a three SNP difference between the *R. ornithinolytica* and the *Enterobacter kobei* plasmid. Based on the published mutation rate for *K. pneumoniae* (2.7×10^−6^ SNPs per site per year, equating to 14 SNPs per genome per year) [61], this would suggest that the plasmids last shared a common ancestor more than 3 years ago. Identification of such resistance plasmids spreading within and between bacterial species is essential when trying to understand the epidemiology of CPE, as has been shown in studies using PacBio sequencing [9, 10]. A study by Sheppard *et al.* [10] particularly emphasizes that a similar detailed picture of plasmid transfer and variability is not available when only studying short-read data, which may prove misleading.

The main limitation of this study is the lack of quantification of CPE load per sample. This was due to technical limitations relating to the incomplete selectivity of currently available CPE media (which results in overgrowth of organisms other than CPE), combined with the low prevalence of CPE in samples. This leads to a low positive predictive value for detection of a true CPE from the growth obtained on the plate and makes quantitation extremely challenging. In order to enrich for low numbers of CPE in a very high bacterial background, we used an additional selective enrichment step (tryptic soy broth with imipenem), which increased the yield of CPE, but ruled out quantitation. Furthermore, the study was conducted in a region with very low CPE prevalence and may not be generalizable across the UK [62]. Future studies including longitudinal data from additional regions and hospitals will provide further insights into the community spread of CPE.

In conclusion, the combined use of Illumina and MinION technologies revealed sharing of plasmids carrying multiple antimicrobial-resistance genes between different bacterial species in wastewater, including the plasmid encoding OXA-48 that confers carbapenem resistance. Whilst phenotypic testing resolved the general class of carbapenemase and short-read data identified the specific carbapenemase genes involved, long-read data was essential to resolve the plasmid architecture and for accurate comparisons. Small, portable sequencing instruments, such as the MinION, have the potential for use in real-time genomic surveillance in a One Health approach that includes genetic material from wastewater, animals and hospitals to monitor the effectiveness of treatment systems, and to contribute to the development of interventions to limit the dissemination of antimicrobial resistance.

## Data bibliography

1. Ludden C, Reuter S, Judge K, Gouliouris T, Blane B *et al.* European Nucleotide Archive, ERS634376 and ERS1033541 (MinION sequence data) (2017).
2. Ludden C, Reuter S, Judge K, Gouliouris T, Blane B *et al.* Github. https://github.com/kim-judge/wastewater (2017).
3. Judge K, Hunt M, Reuter S, Tracey A, Quail, MA *et al.* European Nucleotide Archive, ERS634378 (MinION sequence data) (2016).
4. Judge K, Hunt M, Reuter S, Tracey A, Quail, MA *et al.* European Nucleotide Archive, ERS634378: FKLS01000001– FKLS01000010 (2016).

## Supplementary Data

43 Supplementary File 1

## Supplementary Data

43 Supplementary File 2

## References

[1] Nordmann P, Naas T, Poirel L (2011). Global spread of carbapenemase-producing *Enterobacteriaceae*. Emerg Infect Dis.

[2] Jain A, Hopkins KL, Turton J, Doumith M, Hill R (2014). NDM carbapenemases in the United Kingdom: an analysis of the first 250 cases. J Antimicrob Chemother.

[3] Findlay J, Hopkins KL, Alvarez-Buylla A, Meunier D, Mustafa N (2017). Characterization of carbapenemase-producing Enterobacteriaceae in the West Midlands region of England: 2007–14. J Antimicrob Chemother.

[4] Woodford N, Wareham DW, Guerra B, Teale C (2014). Carbapenemase-producing Enterobacteriaceae and non-Enterobacteriaceae from animals and the environment: an emerging public health risk of our own making?. J Antimicrob Chemother.

[5] Tzouvelekis LS, Markogiannakis A, Piperaki E, Souli M, Daikos GL (2014). Treating infections caused by carbapenemase-producing *Enterobacteriaceae*. Clin Microbiol Infect.

[6] Falagas ME, Tansarli GS, Karageorgopoulos DE, Vardakas KZ (2014). Deaths attributable to carbapenem-resistant *Enterobacteriaceae* infections. Emerg Infect Dis.

[7] Wellington EM, Boxall AB, Cross P, Feil EJ, Gaze WH (2013). The role of the natural environment in the emergence of antibiotic resistance in Gram-negative bacteria. Lancet Infect Dis.

[8] Haller S, Eller C, Hermes J, Kaase M, Steglich M (2015). What caused the outbreak of ESBL-producing *Klebsiella pneumoniae* in a neonatal intensive care unit, Germany 2009 to 2012? Reconstructing transmission with epidemiological analysis and whole-genome sequencing. BMJ Open.

[9] Conlan S, Thomas PJ, Deming C, Park M, Lau AF (2014). Single-molecule sequencing to track plasmid diversity of hospital-associated carbapenemase-producing Enterobacteriaceae. Sci Transl Med.

[10] Sheppard AE, Stoesser N, Wilson DJ, Sebra R, Kasarskis A (2016). Nested Russian doll-like genetic mobility drives rapid dissemination of the carbapenem resistance gene *bla*_KPC_. Antimicrob Agents Chemother.

[11] Judge K, Harris SR, Reuter S, Parkhill J, Peacock SJ (2015). Early insights into the potential of the Oxford Nanopore MinION for the detection of antimicrobial resistance genes. J Antimicrob Chemother.

[12] Ashton PM, Nair S, Dallman T, Rubino S, Rabsch W (2015). MinION nanopore sequencing identifies the position and structure of a bacterial antibiotic resistance island. Nat Biotechnol.

[13] Quick J, Loman NJ, Duraffour S, Simpson JT, Severi E (2016). Real-time, portable genome sequencing for Ebola surveillance. Nature.

[14] Turton JF, Doumith M, Hopkins KL, Perry C, Meunier D (2016). Clonal expansion of *Escherichia coli* ST38 carrying a chromosomally integrated OXA-48 carbapenemase gene. J Med Microbiol.

[15] Oliveira S, Moura RA, Silva KC, Pavez M, Mcculloch JA (2014). Isolation of KPC-2-producing *Klebsiella pneumoniae* strains belonging to the high-risk multiresistant clonal complex 11 (ST437 and ST340) in urban rivers. J Antimicrob Chemother.

[16] Isozumi R, Yoshimatsu K, Yamashiro T, Hasebe F, Nguyen BM (2012). *bla*_NDM-1_-positive *Klebsiella pneumoniae* from environment, Vietnam. Emerg Infect Dis.

[17] Montezzi LF, Campana EH, Corrêa LL, Justo LH, Paschoal RP (2015). Occurrence of carbapenemase-producing bacteria in coastal recreational waters. Int J Antimicrob Agents.

[18] Zurfluh K, Hächler H, Nüesch-Inderbinen M, Stephan R (2013). Characteristics of extended-spectrum β-lactamase- and carbapenemase-producing *Enterobacteriaceae* isolates from rivers and lakes in Switzerland. Appl Environ Microbiol.

[19] Poirel L, Barbosa-Vasconcelos A, Simões RR, da Costa PM, Liu W (2012). Environmental KPC-producing *Escherichia coli* isolates in Portugal. Antimicrob Agents Chemother.

[20] Galler H, Feierl G, Petternel C, Reinthaler FF, Haas D (2014). KPC-2 and OXA-48 carbapenemase-harbouring Enterobacteriaceae detected in an Austrian wastewater treatment plant. Clin Microbiol Infect.

[21] Girlich D, Poirel L, Szczepanowski R, Schlüter A, Nordmann P (2012). Carbapenem-hydrolyzing GES-5-encoding gene on different plasmid types recovered from a bacterial community in a sewage treatment plant. Appl Environ Microbiol.

[22] Picão RC, Cardoso JP, Campana EH, Nicoletti AG, Petrolini FVB (2013). The route of antimicrobial resistance from the hospital effluent to the environment: focus on the occurrence of KPC-producing *Aeromonas* spp. and Enterobacteriaceae in sewage. Diagn Microbiol Infect Dis.

[23] Köser CU, Holden MT, Ellington MJ, Cartwright EJ, Brown NM (2012). Rapid whole-genome sequencing for investigation of a neonatal MRSA outbreak. N Engl J Med.

[24] Zerbino DR, Birney E (2008). Velvet: algorithms for de novo short read assembly using de Bruijn graphs. Genome Res.

[25] Hoffmann H, Roggenkamp A (2003). Population genetics of the nomenspecies *Enterobacter cloacae*. Appl Environ Microbiol.

[26] Zankari E, Hasman H, Cosentino S, Vestergaard M, Rasmussen S (2012). Identification of acquired antimicrobial resistance genes. J Antimicrob Chemother.

[27] Reuter S, Ellington MJ, Cartwright EJ, Köser CU, Török ME (2013). Rapid bacterial whole-genome sequencing to enhance diagnostic and public health microbiology. JAMA Intern Med.

[28] Judge K, Hunt M, Reuter S, Tracey A, Quail MA (2016). Comparison of bacterial genome assembly software for MinION data and their applicability to medical microbiology. Microb Genom.

[29] Li H, Durbin R (2009). Fast and accurate short read alignment with Burrows-Wheeler transform. Bioinformatics.

[30] Li H, Handsaker B, Wysoker A, Fennell T, Ruan J (2009). The sequence alignment/map format and SAMtools. Bioinformatics.

[31] Berlin K, Koren S, Chin CS, Drake JP, Landolin JM (2015). Assembling large genomes with single-molecule sequencing and locality-sensitive hashing. Nat Biotechnol.

[32] Koren S, Walenz BP, Berlin K, Miller JR, Bergman NH (2017). Canu: scalable and accurate long-read assembly via adaptive *k*-mer weighting and repeat separation. Genome Res.

[33] Hunt M, Silva ND, Otto TD, Parkhill J, Keane JA (2015). Circlator: automated circularization of genome assemblies using long sequencing reads. Genome Biol.

[34] Bankevich A, Nurk S, Antipov D, Gurevich AA, Dvorkin M (2012). SPAdes: a new genome assembly algorithm and its applications to single-cell sequencing. J Comput Biol.

[35] Seemann T (2014). Prokka: rapid prokaryotic genome annotation. Bioinformatics.

[36] Page AJ, de Silva N, Hunt M, Quail MA, Parkhill J (2016). Robust high-throughput prokaryote de novo assembly and improvement pipeline for Illumina data. Microb Genom.

[37] Carver TJ, Rutherford KM, Berriman M, Rajandream MA, Barrell BG (2005). ACT: the Artemis comparison tool. Bioinformatics.

[38] Rutherford K, Parkhill J, Crook J, Horsnell T, Rice P (2000). Artemis: sequence visualization and annotation. Bioinformatics.

[39] Bonfield JK, Whitwham A (2010). Gap5—editing the billion fragment sequence assembly. Bioinformatics.

[40] Otto TD, Sanders M, Berriman M, Newbold C (2010). Iterative Correction of Reference Nucleotides (iCORN) using second generation sequencing technology. Bioinformatics.

[41] Poirel L, Héritier C, Tolün V, Nordmann P (2004). Emergence of oxacillinase-mediated resistance to imipenem in *Klebsiella pneumoniae*. Antimicrob Agents Chemother.

[42] Ellington MJ, Kistler J, Livermore DM, Woodford N (2007). Multiplex PCR for rapid detection of genes encoding acquired metallo-β-lactamases. J Antimicrob Chemother.

[43] Mushtaq S, Irfan S, Sarma JB, Doumith M, Pike R (2011). Phylogenetic diversity of *Escherichia coli* strains producing NDM-type carbapenemases. J Antimicrob Chemother.

[44] Yigit H, Queenan AM, Anderson GJ, Domenech-Sanchez A, Biddle JW (2001). Novel carbapenem-hydrolyzing β-lactamase, KPC-1, from a carbapenem-resistant strain of *Klebsiella pneumoniae*. Antimicrob Agents Chemother.

[45] Woodford N, Warner M, Pike R, Zhang J (2011). Evaluation of a commercial microarray to detect carbapenemase-producing Enterobacteriaceae. J Antimicrob Chemother.

[46] Ellington MJ, Findlay J, Hopkins KL, Meunier D, Alvarez-Buylla A (2016). Multicentre evaluation of a real-time PCR assay to detect genes encoding clinically relevant carbapenemases in cultured bacteria. Int J Antimicrob Agents.

[47] Doumith M, Ellington MJ, Livermore DM, Woodford N (2009). Molecular mechanisms disrupting porin expression in ertapenem-resistant *Klebsiella* and *Enterobacter* spp. clinical isolates from the UK. J Antimicrob Chemother.

[48] Zhou K, Yu W, Bonnet R, Cattoir V, Shen P (2017). Emergence of a novel *Enterobacter kobei* clone carrying chromosomal-encoded CTX-M-12 with diversified pathogenicity in northeast China. New Microbes New Infect.

[49] Shin SH, Um Y, Beak JH, Kim S, Lee S (2013). Complete genome sequence of *Raoultella ornithinolytica* strain B6, a 2,3-butanediol-producing bacterium isolated from oil-contaminated soil. Genome Announc.

[50] Poirel L, Bonnin RA, Nordmann P (2012). Genetic features of the widespread plasmid coding for the carbapenemase OXA-48. Antimicrob Agents Chemother.

[51] Poirel L, Potron A, Nordmann P (2012). OXA-48-like carbapenemases: the phantom menace. J Antimicrob Chemother.

[52] Manageiro V, Pinto M, Caniça M (2015). Complete sequence of a *bla*_OXA__-48_-harboring IncL plasmid from an *Enterobacter cloacae* clinical isolate. Genome Announc.

[53] Power K, Wang J, Karczmarczyk M, Crowley B, Cotter M (2014). Molecular analysis of OXA-48-carrying conjugative IncL/M-like plasmids in clinical isolates of *Klebsiella pneumoniae* in Ireland. Microb Drug Resist.

[54] Al-Bayssari C, Olaitan AO, Leangapichart T, Okdah L, Dabboussi F (2016). Whole-genome sequence of a *bla*_OXA-48_-harboring *Raoultella ornithinolytica* clinical isolate from Lebanon. Antimicrob Agents Chemother.

[55] Carattoli A, Bertini A, Villa L, Falbo V, Hopkins KL (2005). Identification of plasmids by PCR-based replicon typing. J Microbiol Methods.

[56] Chen YT, Liao TL, Liu YM, Lauderdale TL, Yan JJ (2009). Mobilization of *qnrB2* and IS*CR1* in plasmids. Antimicrob Agents Chemother.

[57] Water UK (2014). *National Guidance for Healthcare Waste Water Discharges*; issue date August 2014.. https://dldropboxusercontentcom/u/299993612/Publications/Guidance/Wastewater/Water.

[58] Hocquet D, Muller A, Bertrand X (2016). What happens in hospitals does not stay in hospitals: antibiotic-resistant bacteria in hospital wastewater systems. J Hosp Infect.

[59] Uz Zaman T, Aldrees M, Al Johani SM, Alrodayyan M, Aldughashem FA (2014). Multi-drug carbapenem-resistant *Klebsiella pneumoniae* infection carrying the OXA-48 gene and showing variations in outer membrane protein 36 causing an outbreak in a tertiary care hospital in Riyadh, Saudi Arabia. Int J Infect Dis.

[60] Carattoli A, Seiffert SN, Schwendener S, Perreten V, Endimiani A (2015). Differentiation of IncL and IncM plasmids associated with the spread of clinically relevant antimicrobial resistance. PLoS One.

[61] Chung The H, Karkey A, Pham Thanh D, Boinett CJ, Cain AK (2015). A high-resolution genomic analysis of multidrug-resistant hospital outbreaks of *Klebsiella pneumoniae*. EMBO Mol Med.

[62] Trepanier P, Mallard K, Meunier D, Pike R, Brown D (2017). Carbapenemase-producing Enterobacteriaceae in the UK: a national study (EuSCAPE-UK) on prevalence, incidence, laboratory detection methods and infection control measures. J Antimicrob Chemother.

