# Supplementary material for: Sharing of carbapenemase-encoding plasmids between Enterobacteriaceae in UK sewage uncovered by MinION sequencing
Source: Microb Genom. 2017 Jul 4;3(7):e000114. doi: 10.1099/mgen.0.000114 (PMC5605956; doi:10.1099/mgen.0.000114)
Supplement: Supplementary File 2 [file mgen-3-114-s002.pdf]

Supplemental Table S2.

	Number of reads	Average read size	N50 read size	Largest read
VRES0259 (pass)	3782	2980	5726	23822
VRES0259 (fail)	148594	2825	6115	962097
VRES0259 (all)	152376	2829	6109	962097
VRES0269 (pass)	30970	4832	7373	53435
VRES0269 (fail)	43193	3855	6176	242451
VRES0269 (all)	74163	4424	7013	242451
VRES0273 (pass)	43260	5357	7032	42042
VRES0273 (fail)	21237	3768	6878	420073
VRES0273 (all)	64497	4834	6997	420073
